# Bedtime routines child wellbeing & development

**DOI:** 10.1186/s12889-018-5290-3

**Published:** 2018-03-21

**Authors:** George Kitsaras, Michaela Goodwin, Julia Allan, Michael P. Kelly, Iain A. Pretty

**Affiliations:** 1 Dental Health Unit, Division of Dentistry, The University of Manchester, Manchester, UK; 2 Institute of Applied Health Sciences, University of Aberdeen, Aberdeen, UK; 3 Department of Public Health and Primary Care, University of Cambridge, Cambridge, UK

**Keywords:** Child, Bedtime, Parenting, School readiness, Dental caries, Executive function

## Abstract

**Background:**

Bedtime routines has shown important associations with areas associated with child wellbeing and development. Research into bedtime routines is limited with studies mainly focusing on quality of sleep. The objectives of the present study were to examine the relationship between bedtime routines and a variety of factors associated with child wellbeing and to examine possible determinants of bedtime routines.

**Methods:**

A total of 50 families with children between 3 and 5 years old took part in the study. Data on bedtime routines, parenting styles, school readiness, children’s dental health, and executive function were collected.

**Results:**

Children in families with optimal bedtime routines showed better performance in terms of executive function, specifically working memory (t (44)= − 8.51, *p* ≤ .001), inhibition and attention (t (48)= − 9.70, *p* ≤ .001) and cognitive flexibility (t (48)= − 13.1, *p* ≤ .001). Also, children in households with optimal bedtime routines scored higher in their readiness for school (t (48)= 6.92, *p* ≤ .001) and had better dental health (U = 85.5, *p* = .011). Parents in households with suboptimal bedtime routines showed worse performance on all measures of executive function including working memory (t (48)= − 10.47, *p* ≤ .001), inhibition-attention (t (48)= − 10.50, *p* ≤ .001) and cognitive flexibility (t (48)= − 13.6, *p* ≤ .001). Finally, parents with optimal bedtime routines for their children deployed a more positive parenting style in general (i.e. authoritative parenting) compared to those with suboptimal bedtime routines (t (48)= − 6.45, *p* ≤ .001).

**Conclusion:**

The results of the present study highlight the potentially important role of bedtime routines in a variety of areas associated with child wellbeing and the need for further research.

**Electronic supplementary material:**

The online version of this article (10.1186/s12889-018-5290-3) contains supplementary material, which is available to authorized users.

## Background

Public Health England (PHE) classifies wellbeing as: “*mental and physical health elements incorporating emotional, social and developmental aspects along perceived satisfaction and optimal quality of life”* [1]. A limited number of existing studies have shown important associations between bedtime routines and a number of factors linked to child development, child wellbeing and parenting [2–6]. Quality of sleep, dental health, school performance including school readiness, socio-emotional and cognitive development have shown important, yet in some cases limited, associations with bedtime routines. These factors can directly affect components associated with overall wellbeing including mental and physical health as well as emotional, social and developmental aspects while impacting perceived satisfaction and quality of life resulting in direct [1, 2, 4–7].

Overall, bedtime routines and quality of sleep has attracted the most research interest with studies consistently showing that better quality bedtime routines are associated with better sleep quality and duration for both adults and children [6–8]. Other studies have highlighted the importance of bedtime routines in developing a healthy attitude towards learning, reading and ultimately school [2]. Children who read regularly with their parents as part of their bedtime routine (or are read to by their parents) show improvements in language, reading and literacy rates as well as better school readiness [2, 9]. School readiness, closely associated with a healthy attitude towards school, has impact beyond the first years of school education with children who have higher levels of school readiness at age five presenting generally with more successful grades at school, being less likely to drop out of high school and even earn more as adults [9]. Finally, associations have been found between bedtime routine patterns of brain development, and socio-emotional skills development and a stronger parent-child relationship [10].

Another area closely associated with bedtime routines is dental health. Oral hygiene behaviours (brushing teeth, avoiding snacks before bed) as part of bedtime routines have shown significant correlations with improved oral health with lower prevalence of caries (decay) in both children and adults [2, 11, 12]. On the contrary, children whose parents allow them to consume products rich in sugars during bedtime routines show higher levels of caries compared to children whose parents have a more robust routine in place [5]. Poor dental health during childhood can have a negative impact on the life of preschool children and their parents [13]. From a wider health perspective, the negative impact of dental caries in an early age includes chewing difficulties, decreased appetite, weight loss, sleeping difficulties, changes in behaviour (such as irritability), implications for psychological development (with low self-esteem having been suggested) and decrease in school performance [13–15]. Additionally, dental problems in young children possess a significant financial expense for families with high direct and indirect costs [16]. Additionally, in many cases dental caries require hospitalization and especially visits to emergency departments that can be extremely stressful for parents and frightening for children [16]. Untreated dental disease in children increases their risk for dental extraction under general anaesthetic a process that has significant impact on children and their families and it can increase the risk of dental anxiety [17]. Apart from child and family related implications dental caries also impact upon public finances and function of healthcare systems around the globe with US$298 billion (or 4.6% of global health expenditure) spent on direct dental treatments alone [18].

Finally, bedtime routines have been associated with emotional and psychological wellbeing in parents and children. Children with non-regular bedtime routines experience more frequent behavioural difficulties than others [4] and parents with optimal bedtime routines report lower levels of anxiety, anger and fatigue [19]. Research on family routines in general demonstrates the importance of parent-related as well as child-related factors (parental self-regulation, parental efficacy, parenting practices, socio-emotional wellbeing, parent-child relationship) in allowing positive routines to be developed and established [10]. Consistent and beneficial routines are essential for positive child development and family functioning and can expose the extent of affirmative and negative parenting practices within a family [10]. Routines also have important associations with parent-child dynamics and overall family functioning [20].

### “Gold standard” of bedtime routines

Despite the reported likely importance of bedtime routines, no clear statement or policy on what constitutes an optimal bedtime routine exists. Based on available studies and limited guidelines from different organisation and professional bodies, an optimal bedtime routine for infants and preschool children is likely to; be consistent throughout the week and weekend, follow the recommended sleep times for each age group (i.e.10–13 h of sleep, including naps, for children between 3 and 5 years of age etc.), include tooth brushing and avoidance of drinks (such as bottle feeding) and snacks before bed, minimise the use of electronic devices and television around and during bedtimes, consider a bath or shower before bed and finally, include book reading and book sharing activities before sleep [6–8, 19, 21–23].

As optimal bedtime routines have multiple potentially beneficial components the cumulative effect of all of them can result in multiple positive outcomes and wider benefits for wellbeing and development. However, the majority of research in this area focuses on just one beneficial outcome of routines – sleep quality. That focus is creating a gap in our understanding that needs to be addressed by more inclusive studies that move away from quality of sleep and examine other areas associated with wellbeing and development as highlighted by a review from Mindell & Williamson [24].

### Objectives

The principle objective of this study was to investigate if bedtime routines are associated with a diverse range of key indicators of child wellbeing and development. More specifically, the study investigated whether optimal bedtime routines are associated with (a) greater readiness for school, (b) better dental health and (c) higher executive function in preschool age children. These three areas of child health and wellbeing were selected due to their important associations with further child development, overall wellbeing, achievement and impact on quality of life. Moreover, prior research into bedtime routines heavily focused on quality of sleep as its primary objective with only a limited number of studies incorporating additional measurements of health, wellbeing and development. Additionally, an examination of possible determinants of bedtime routines formed the secondary objective of the study by exploring whether optimal routines are more likely in families where parents have (a) higher executive function and (b) positive parenting styles.

## Methods

### Participants

One parent and one child from each of fifty (50) families took part in the study. Parents had a mean age of 35 years (SD = 5) and were, as expected in research involving families, predominantly female (78%). Most (70%) had no university-level education and were either part-time employed (19%) or stay at home parents (21%). Children had a mean age of 4 years (SD = 0.8 months) and were relatively evenly split by gender (48% male, 52% female). Socio-demographic characteristics of the sample represented the overall demographic composition of the area where the study was conducted with 66% White, 18% Asian and 16% Black in terms of ethnicity. Finally regarding their socio-economic background, based on the Index of Multiple Deprivation (IMD) where higher quintiles represent higher deprivation, 10% of the sample came from the 2nd quintile, 22% from the 3rd, 26% from the 4th and 42% from the 5th quintile respectively. There were no refusals to participate in the study and no withdrawals throughout the duration of data collection. Additional file 1 contains full characteristics of the sample.

### Recruitment

During February to May 2017, participants were recruited in the study: (a) through an active study on General Dental Anaesthetic teeth extraction who had expressed interest in participating in future studies and (b) through General Dental Practices (GDPs). Two selection criteria were present during recruitment: (a) having children between the ages of 3 and 5 and (b) sufficient English literacy to provide informed consent and complete questionnaires and assessments. During recruitment, information leaflets for both adults and children were provided; parents completed consent forms while child assent was sought throughout the process in order to ensure the willingness of each child to participate in the study. Parents were the ones initially approached at GDPs resulting in researchers not being aware of each child’s attendance to regular dental appointments.

### Data collection process

Data collection took place between March and June 2017, while recruitment was underway, either in house visit, in the dental practices where the families were recruited or in a neutral venue. Two visits/meetings were necessary in order to complete the data collection with the first visit/meeting comprising of the parent-related assessment and the second meeting including the child-related assessments. In between the two visits/meetings, parents were sent a 5 nightlong interactive text questionnaire to assess the quality of each night’s bedtime routines. Each participating family received a total of £50 in shopping vouchers as compensation for their time.

### Measurements

#### Bedtime routines

All available measures of bedtime routines utilise a retrospective design with many of them containing a long-list of questions with increase risk of recall bias. The present study, following Patient Public Involvement (PPI) work, opted for the development of an interactive text based survey for the assessment of bedtime routines.

**Fig. 1:**
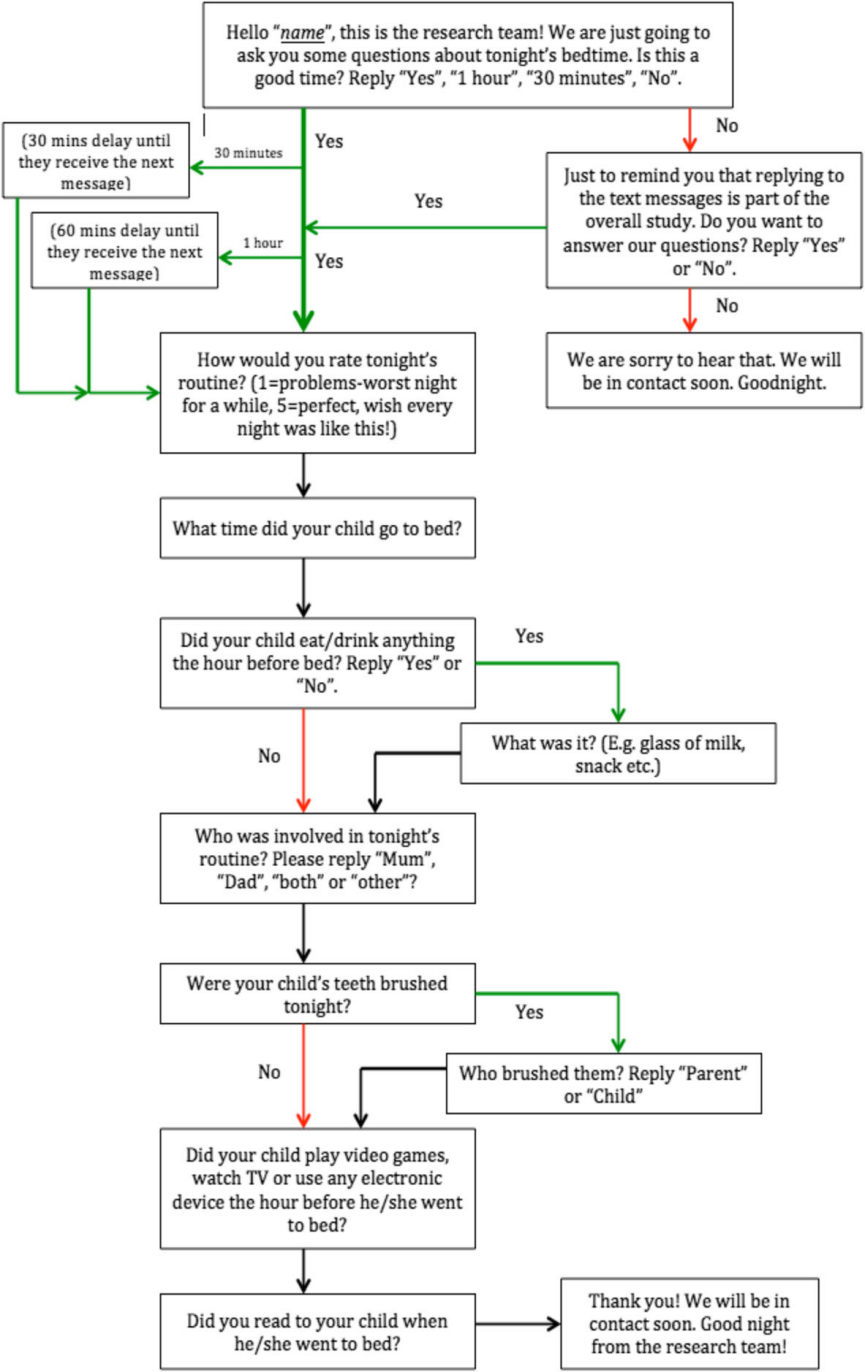
Branching logic of text survey bedtime routines assessment. For 5 consecutive nights each participating family received the same initial text message (Hello “name”….). Depending on their responses they continued to receive text messages until they reached the end of the survey

Focusing on the areas previously identified from the literature as being components of optimal bedtime routines, the interactive assessment focused on 5 target areas: a. consistency (determined as child going to bed within the space of an hour every night), b. tooth brushing, c. avoidance of snack/drinks before bed, d. avoidance of electronic devices before bed and e. book reading (see Fig. 1 for the branching logic of the text-survey). The interactive text-survey was sent for 5 consecutive nights directly to participating parents’ mobile phones. Questions were both open-ended (e.g. “What did the child eat before bed?”) and closed (e.g. “How would you rate tonight’s routine from 1 (=problems, worst night for a while) to 5(=perfect, wish every night was like this!”). Consistency of implemented bedtime routines was monitored by a closed question (Who was involved in tonight’s routine? Please specify “Mum”, “Dad”, “Both” or “Other”).

A score of 1 was assigned for each of components of the bedtime routines parents reported on a nightly basis. For example a family that reported brushing teeth, avoiding snacks and reading a book before bed received a score of 3 out of 5 for that night. Average scores for the 5 nights were calculated and used for further analyses. High scores indicate better bedtime routines. Cumulative, rather than separate component-based scores, were utilised in order to better understand the overall impact of bedtime routines as with previous research [25]. Effects of social desirability bias were considered as a possible limitation. However, the design of the assessment with its fast pace, short and direct questions and the administration over a 5 night period were considered important counter-measures to minimise the effect of this type of bias.

#### School readiness

School readiness was assessed using the Bracken School Readiness Assessment-3rd Edition (BSRA-3) [26]. BSRA-3 consists of 88 items/tasks for children to complete under instruction, measuring concepts such as colours, number/counting, letters, size/comparison and shapes. The task takes approximately 15 min to administer and is suitable for children aged between 3 years and 6 years and 11 months. The BSRA-3 is a nonverbal task, minimising interference from language development, and it determines if a child is ready for school [26]. BSRA-3 has good test-retest reliability across time for all age groups (.76 to .92) and its internal consistency with split-half reliability is excellent (.95). High scores indicate greater readiness for school. Assessment of school readiness across all children participating in the study, even for the ones already in school, is crucial since many children in the first year of primary school still present as not school ready [27].

#### Child dental health

Child dental health was objectively assessed through dmft (decayed, missing or filled primary teeth) scores assigned either through examination of dental charts, history of extraction of carious teeth under general anaesthetic or by a registered dentist. Dmft scores are amongst the most commonly used methods in oral epidemiology for assessing dental caries prevalence and overall dental health. Higher dmft scores indicate poorer dental health.

#### Executive function

Executive function was assessed by two separate assessments. First, executive function for both adults and children was assessed using the National Institute of Health (NIH) Toolbox for the assessment of neurological and behavioural function (NIH-Toolbox) [28]. All assessments were administered electronically through an iPad. Three [3] neuropsychological assessments of executive function were selected focusing on attention/inhibition (=Flanker Inhibitory Control and Attention Task), working memory (=List Sorting-Working Memory Task) and cognitive flexibility/shifting (=Dimensional Change Card Sort) representing the three main domains of executive function [29]. Higher scores in the NIH-Toolbox assessment indicate better executive functioning.

Perceived executive functioning in day to day life was assessed using the Behavioural Rating Inventory of Executive Function (BRIEF) with versions focusing on adults (18+) (BRIEF-A) and preschool [3–5] (BRIEF-P) [30]. BRIEF-A is composed of 75 items. Both BRIEF-P and BRIEF-A have appropriate internal consistency and temporal stability with α = 0.97 and α = 0.96 for the composite score in the BRIEF-P and BRIEF-A respectively. Low scores indicate better executive functioning (i.e. less dysfunction).

#### Parenting styles

The short-version of the Parenting Style and Dimensions Questionnaire (PSDQ***)*** [31] was administered to assess parenting styles. The PSDQ produces scores in three parenting styles: authoritative, authoritarian and permissive with underlying sub-dimensions. The score for each sub-dimension is calculated on the mean of all items within the sub-dimension. Each parenting style is calculated by taking the mean of the scores for the sub-dimensions within each style. The authors reported internal consistency reliabilities (Cronbach alphas) for mothers’ and fathers’ reports to be .86 (authoritative), .82 (authoritarian), and .64 (permissive).

### Statistical analysis

All paper-based measurements (BRIEF-A/P, PSDQ, BSRA-3) were scored following the official professional manuals of the developers. BRIEF-A/P uses T scores (M = 50, SD = 10) that are transformations of the raw scale scores. BSRA-3 uses standard scores based on the age of the child and his/her raw score. Age-corrected scores (M = 100, SD = 15) from the neuropsychological assessment (NIH-Toolbox) were automatically calculated. Based on the 0–5 scales used to score bedtime routines, families with scores between 0 and 2 were coded as having “suboptimal bedtime routines” while families with scores between 3 and 5 were coded as having “optimal bedtime routines”. All data were entered into SPSS version 22 [32].

Bivariate correlations (Pearson’s r) were conducted between standardised metrics collected for the study (BSRA-3 for school readiness, BRIEF-A/P & NIH-Toolbox for executive function and PSDQ for parenting) to ensure that, as with previous studies, these measurements present strong inter-correlations that will allow for subsequent analyses. Between groups comparisons (independent sample t-test) were conducted for optimal and sub-optimal bedtime routines while a Mann-Whitney U test was conducted to examine differences in dental health between children with optimal and suboptimal bedtime routines.

## Results

### Comparisons based on bedtime routines for parenting, executive function and school readiness

Table 1 shows there were significant differences across all metrics. Positive (i.e. authoritative parenting) was more common in household with optimal (M = 3.6, SD = 0.68) than suboptimal (M = 2.3, SD = 0.72) bedtime routines, t (48)= − 6.45, *p* ≤ .001. Negative parenting including both authoritarian and permissive parenting styles was more common in households with suboptimal (M = 2.5, SD = 0.57) (authoritarian) / (M = 3.3., SD = 0.97) (permissive) than optimal (M = 1.5, SD = 2.5) (authoritarian) / M = 1.9, SD = 0.54) (permissive) bedtime routines, t (48)= 6.50, *p* ≤ .001 for authoritarian and t (48)= 5.82, *p* = .003 for permissive parenting. Children presented as less ready for school with lower scores of school readiness in households with suboptimal (M = 81.2, SD = 5.4) rather than optimal (M = 106.1, SD = 8.00) bedtime routines, t (48)= − 12.15, *p* ≤ .001.

**Table 1:** Differences on parenting, executive function and school readiness between families with optimal and suboptimal bedtime routines

		Optimal Bedtime Routines (M, SD)	Sub-optimal Bedtime Routines (M, SD)	df	Sig. (2-tailed)	Mean Difference (95% C.I.)
Parenting	Authoritative Parenting	3.4 (0.68)	2.3 (0.72)	48	.001^a^	−1.313 (−1.721, −.904)
	Authoritarian Parenting	1.5 (2.50)	2.5 (0.57)	48	.001^a^	1.040 (.719, 1.362)
	Permissive Parenting	1.9 (0.54)	3.3 (0.97)	48	.003^b^	1.392 (.9000, 1.882)
Executive Function / Adults	Inhibition-Attention Adults	102.7 (7.00)	83.7 (4.90)	48	.001^a^	−18.983 (−22.615,-15.351)
	Working Memory Adults	100.1 (6.13)	80.4 (7.05)	48	.001^a^	−19.650 (−23.431, − 15.868)
	Cognitive Flexibility Adults	105.3 (5.59)	84.3 (4.96)	48	.001^a^	−21.017 (−24.123, −17.910)
	BRIEF-Adults (Global Executive Composite)^c^	45.3 (3.59)	53.1 (2.51)	48	.007^a^	7.800 (5.938, 9.662)
	School Readiness	106.1 (8.00)	81.2 (5.40)	48	.001^a^	−24.900 (−29.020, −20.780)
Executive Function / Children	Inhibition-Attention Children	103.2 (5.59)	84.3 (7.29)	48	.001^a^	−18.850 (−22.753, −14.946)
	Working Memory Children	100.3 (6.25)	83.0 (7.29)	44	.001^a^	−17.310 (−21.406, − 13.213)
	Cognitive Flexibility Children	105.1 (5.74)	84.1 (5.25)	48	.001^a^	−21.066 (−24.289, −17.843)
	BRIEF-Preschool Children (Global Executive Composite)^c^	42.7 (2.88)	48.3 (2.61)	48	.005^a^	5.566 (3.950, 7.182)

^a^ Equal variances assumed

^b^ Equal variances not assumed

^c^ Lower scores indicate better executive function

As for executive function, in the case of adults and regarding the assessments performed using the neuropsychological assessment, poorer performance on the inhibition and attention task was found in parents of households with suboptimal (M = 83.7, SD = 4.9) than optimal (M = 102.7, SD = 7.00) bedtime routines, t (48)= − 10.50, *p* ≤ .001. Also, parents with suboptimal bedtime routines scored lower in the working memory task (M = 80.4, SD = 7.05) than those with optimal routines (M = 100.1, SD = 6.13), t (48)= − 10.47, *p* ≤ .001. Finally, lower scores of cognitive flexibility were observed in parents reporting suboptimal (M = 84.3, SD = 4.96) than optimal (M = 105.3, SD = 5.59) bedtime routines, t (48)= − 13.6, *p* ≤ .001. In the case of children, lower scores in the attention and inhibition task were present in suboptimal (M = 84.3, SD = 7.29) than optimal (M = 103.2, SD = 7.49) bedtime routines, t (48)= − 9.70, *p* ≤ .001. Also, children from households with suboptimal bedtime routines showed poorer performance in working memory (M = 83.0, SD = 7.29) compared to those from households with optimal (M = 100.3, SD = 6.25) bedtime routines, t (44)= − 8.51, *p* ≤ .001. Finally, children with suboptimal bedtime routines showed less cognitive flexibility (M = 84.1, SD = 5.25) than those with optimal (M = 105.1, SD = 5.74) bedtime routines, t (48)= − 13.1, *p* ≤ .001. Regarding self-reported executive functioning (BRIEF-A & BRIEF-P), both parents and children in households with suboptimal bedtime routines scored higher (M = 53.1, SD = 2.51) (adults) / (M = 48.3, SD = 2.61) (children) – indicating poorer executive function- compared with those in households with optimal bedtime routines (M = 45.3, SD = 3.59) (adults) / (M = 42.7, SD = 2.88) (children), t (48)= 8.42, *p* ≤ .001 for adults and t (48)= 6.92, *p* ≤ .001 for children.

### Comparisons based on bedtime routines for children’s dental health

The Mann-Whitney test indicated that children in families with optimal bedtime routines presented with better dental health, i.e. less decay and fewer missing or filled teeth (dmft = 0) (Mdn = 4) compared to children in families with suboptimal bedtime routines (dmft > 0) (Grand Mdn = 2), U = 85.5, *p* = .011.

### Associations between metrics

Table 2 presents the results of the bivariate correlations. In general, across all metrics, significant correlations exist in the directions expected. Interestingly, and contrary to available research that observed at best moderate correlations, there were strong correlations between the self-reported executive function inventory and objective neuropsychological assessments of both adults and children. Negative correlations are expected due to the nature of the scores obtained between the two assessments. In children, there was a strong negative significant correlation between the Global Executive Composite of BRIEF-P and inhibition and attention of the neuropsychological assessment, r(50) = −.610, *p* ≤ .001, the Global Executive Composite of BRIEF-P and working memory of the neuropsychological assessment, r (46)= − .575, *p* ≤ .001 and Global Executive Composite of BRIEF-P and cognitive flexibility of the neuropsychological assessment, r(50) = −.639, *p* ≤ .001. Also, in the case of adults, there was a strong negative significant correlation between the Global Executive Composite of BRIEF-A and inhibition and attention of the neuropsychological assessment, r(50) = −.654, *p* ≤ .001, the Global Executive Composite of BRIEF-A and working memory of the neuropsychological assessment, r(50) = − 636, *p* ≤ .001 and the Global Executive Composite of BRIEF-A and cognitive flexibility of the neuropsychological assessment, r(50) = −.656, *p* ≤ .001.

**Table 2:** Associations between school readiness, executive function & parenting

		School readiness	Executive Function / Adults				Parenting			Executive Function / Children			
			Inhibition Attention	Working memory	Cog. flexibility	GEC^a^	Authoritative Parenting	Authoritarian Parenting	Permissive Parenting	Inhibition Attention	Working memory	Cog. flexibility	GEC^a^
School readiness		1											
Executive Function / Adults	Inhibition Attention	.819^b^	1										
	Working memory	.755^b^	.932^b^	1									
	Cognitive flexibility	.795^b^	.933^b^	.945^b^	1								
	GEC^a^	−.629^b^	−.610^b^	−.575^b^	−.639^b^	1							
Parenting	Authoritative Parenting	.602^b^	.645^b^	.608^b^	−.683^b^	−.715^b^	1						
	Authoritarian Parenting	−.624^b^	−.608^b^	−.519^b^	−.631^b^	.560^b^	−.615^b^	1					
	Permissive Parenting	−.611^b^	−.465^b^	−.464^b^	−.540^b^	.621^b^	−.649^b^	.623^b^	1				
Executive Function / Children	Inhibition Attention	.519^b^	.783^b^	.733^b^	.548^b^	−.635^b^	.664^b^	−.608^b^	−.462^b^	1			
	Working memory	.569^b^	.636^b^	.686^b^	.553^b^	−.603^b^	.649^b^	−.593^b^	−.496^b^	.942^b^	1		
	Cognitive flexibility	.407^b^	.641^b^	.541^b^	.590^b^	−.657^b^	.705^b^	−.627^b^	−.552^b^	955^b^	.953^b^	1	
	GEC^a^	−.540^b^	−.408^b^	−.572^b^	−.665^b^	.661^b^	−.563^b^	.558^b^	−.474^b^	−.654^b^	−.636^b^	.656^b^	1

^a^Global Executive Composite of BRIEF-A/P assessment

^b^ Correlation is significant at the .001 level (2-tailed)

School readiness was significantly correlated with better executive function in children with r(50) = .819, *p* ≤ .001 for inhibition and attention, r (46)=.755, *p* ≤ .001 for working memory and r(50) = .795, *p* ≤ .001 for cognitive flexibility indicating that better executive function was related to greater readiness for school. Adults with better EF exhibited more positive parenting with authoritative parenting highly positively correlated with all executive function metrics for adults with r(50) = .664, *p* ≤ .001 for inhibition and attention, r(50) = .649, *p* ≤ .001 for working memory and r(50) = .705, *p* ≤ .001 for cognitive flexibility. Conversely, poor performance on executive function measurements was associated with more negative parenting practices (i.e. authoritarian and permissive parenting) with r(50) = −.608, *p* ≤ .001 for attention/inhibition, r(50) = −.593, *p* ≤ .001 for working memory and r(50) = −.627, *p* ≤ .001 for cognitive flexibility and r(50) = −.462, *p* ≤ .001 for attention/inhibition, r(50) = −.469, *p* ≤ .001 for working memory and r(50) = −.552, *p* ≤ .001 for cognitive flexibility for authoritarian and permissive parenting respectively. Finally, higher parent executive function scores were associated with higher child executive function scores across all metrics of executive function with r(50) = .783, *p* ≤ .001 for attention/inhibition, r(50) = .686, *p* = .001 for working memory and r(50) = .590, *p* ≤ .001 for cognitive flexibility.

## Discussion

The present study attempted to explore the effects of bedtime routines on child wellbeing and development and their association with parenting and executive function. Overall, results indicated the optimal routines were associated with better dental health, cognitive function and school readiness in children and that optimal routines were more likely to be present in households where parents were authoritative in style and had good executive function. Many of the findings from this study are unique while others follow existing observations from previous studies in the field.

### Effect of bedtime routines on child wellbeing and child development

The between group comparisons based on the quality of bedtime routines (optimal or suboptimal) resulted in highly significant differences across all metrics associated with child wellbeing and child development. For school readiness the results of the present study echoed recommendations and findings from previous studies where children with suboptimal routines, including absence of activities like book sharing and book reading, presented with lower school readiness a metric closely associated with subsequent school performance and academic achievement [2, 9, 33]. Another area where the findings of the study are consistent with previous studies is in relation to dental health. Consistently with previous studies, children with suboptimal bedtime routines, including in some cases absence of brushing teeth and/or consuming snacks before bed, presented with worse dental health (dmft > 0) compared to children whose routines included tooth brushing and no snacks before bed (dmft = 0). The findings of the present study follow similar research in the field where children with robust routines that included dental health behaviours like teeth brushing and avoidance of late night snacks showed lower levels of caries and generally more improved dental health [1, 4, 11, 34].

Finally, with regards to the observed differences in executive function between children with optimal and suboptimal bedtime routines, with the former presenting better scores across all metrics of executive function, this finding is new. Previous studies on the development and manifestation of executive function focused primarily on brain development and cortical changes with factors such as deprivation, environmental factors, parenting and sleep [3, 35–38]. Executive function, a complex and highly interdependent group of cognitive processes, develops at an unprecedented rate during the preschool period and it is open to multiple influences [39, 40]. The complex nature of executive function and the myriad factors associated with their development make it difficult to draw firm conclusions from the present results, but the differences in executive function between children with optimal and suboptimal routines are large highlighting the need for further investigation. Sleep, directly affected by the quality of bedtime routines, can be a potentially significant mediator in the observed differences however; quality of sleep was not assessed in the present study potentially limiting our understanding. Moreover, parents with good executive function will be potentially more likely to have children with good executive function due to genetic and biological underpinnings given evidence of high heritability regarding executive function [41]. Based on the apparent importance of bedtime routines for child development and wellbeing, it is vital to gain a better understanding while also considering possible future early interventions to support those with suboptimal routines.

### Parenting styles and parental executive function as possible determinants of the quality of bedtime routines

Between group comparisons based on the quality of bedtime routines showed significant differences in parenting styles and parent executive function between parents with suboptimal bedtime routines as compared to those with optimal bedtime routines. Parents with optimal bedtime routines systematically scored better on executive function tasks including inhibition/attention, working memory and cognitive flexibility and their parenting style was also consistently more authoritative as compared to parents with suboptimal bedtime routines. The differences in executive function between parents with optimal and suboptimal routines can be the result of the previously known relationship between executive functioning and parenting styles [42, 43] where the lower the executive function the more negative the parenting practices, and vice versa, with subsequent implications for the quality of family routines, bedtime routines included. Moreover, it is possible that executive function has a more direct relationship with bedtime routines given the multiple skills associated with that time-period in each family’s day. All elements of executive function are vital for bedtime routines including working memory, that needs to be updated to maintain and manipulate information, attention, crucial in maintaining focus and guiding reactions, inhibition, to control impulsive behaviours and control negative emotions and finally shifting, to switch attention across multiple areas and situational demands [35, 43]. During bedtime, it can be hypothesised, that parents need to exercise the full extent of their cognitive and behavioural regulation capacities in order to achieve an optimal routine in an acceptable timeframe.

Since this is the first time that different parenting styles and parent executive function are examined in relation to their effect on the quality of bedtime routines and that the present data are cross sectional, no firm conclusions can be made. It is important to further examine the stability of the observed, significant, differences in parenting styles and executive function with regards to optimal and suboptimal routines with the inclusion of other important parameters. A series of questions arise from the findings of this study and future studies will need to better understand the very nature of bedtime routines and how they are shaped. Are bedtime routines a direct product of parenting styles and practices with parent executive function serving as a possible mediator of that relationship or vice versa? If bedtime routines change, given that bedtime routines are behaviours repeated over a period of time, will the changes affect parenting and parent executive function? Finally, with changes in parenting styles and practices and even improvements in executive function, despite its inherit decline over time due to aging, will changes in bedtime routines occur?

### Children’s executive function & school readiness

Consistently with existing literature in the field, school readiness scores were strongly positively associated with better executive function scores in the cognitive assessment and strongly negatively associated with the parent-completed inventory. The three aspects of executive function (working memory, inhibition/attention and cognitive flexibility) assessed during the study are considered fundamental for school readiness [44]. Greater ability in cognitive flexibility, attention, inhibition and working memory is crucial in self-regulation and subsequently in allowing children to organise their thinking, minimise reactivity, increase social competence and ultimately support early learning, school readiness and school achievement [45–47]. School readiness is a multi-faceted construct with influences from a variety of factors including socio-economic status, parenting practices with executive function being one of them [36].

### Parent & child executive function and the role of parenting styles

At the moment, mixed and relatively limited results exist with regards to early childhood executive functioning and its association with parental executive function with some studies reporting strong associations that faded over time as the child grew [48]. In our study, parents and children presented strong correlations across all different metrics of executive function in both the neuropsychological assessments and the self-report inventories. Previous studies have shown that executive function might be affected by continuous social exposure with family environment offering daily opportunities for the child to improve and challenge their executive functioning [49]. Therefore, parents with lower executive function can directly and indirectly affect those crucial opportunities for the child to explore and practice its executive function leading to possible subsequent decreased executive function compared to children whose parents have higher executive function. With previous mixed results the present findings contribute to on-going attempts to gain a better understanding of the complex nature of the development and manifestation of executive functioning in preschool age children.

Finally, and in full accordance with previous studies in the field, positive parenting (i.e. an authoritative parenting style) was strongly positively associated with higher executive function. Moreover, negative parenting styles (i.e. authoritarian and permissive) were significantly negatively correlated with executive functioning showing that the parents with worse executive function were more inclined to manifest authoritarian and/or permissive parenting styles and vice versa. The importance of executive function on parenting styles and practices is not difficult to understand when taking into consideration different aspects associated with both elements.

### Limitations

Despite the significant, and in areas unique, findings of the present study some limitations exist. The main limitation of the study is its cross-sectional design that did not account for all possible confounders resulting in constrains regarding our understanding of the potentially causal relationships between the variables measured. Another limitation is the lack of information on potentially important metrics such as: child’s psychosocial development, parental psychological wellbeing and child’s quality of sleep that can have direct and/or indirect effects on observed associations. With regards to the latter, quality of sleep is associated with a number of social-emotional, cognitive, physical health and family functioning domains therefore future studies will need to account for it in order to be able to present an inclusive and holistic picture of all possible associations.

## Conclusion

The main objective of the present study was to explore if bedtime routines affect child wellbeing as measured by dental health, school readiness and executive function. Additionally, the role of parenting and parental executive function as possible determinants of the quality of bedtime routines was examined. Following the analyses, all available findings point to the importance of routines with regards to child wellbeing with significant differences in key metrics between children who have optimal and suboptimal bedtime routines. Moreover, positive parenting and better executive function were both significantly associated with optimal routines allowing for a better understanding in the complex nature of mechanisms involved in the establishment and manifestation of bedtime routines. The present study showed important associations of bedtime routines with a variety of health-related metrics involved in child wellbeing and their relationship with parent-related factors. Despite limitations, the results of this study are unique in the literature, showing the need for more in-depth exploration of bedtime routines given their potentially crucial role in child wellbeing and their association with parent-related factors.

## Additional file

Additional file 1: Descriptive statistics of sample. Sample characteristics including gender, age, educational level, Index of Multiple Deprivation scores, ethnicity and employment status for all participants. (DOCX 20 kb)
