# Supplementary material for: Bedtime routines child wellbeing & development
Source: BMC Public Health. 2018 Mar 21;18:386. doi: 10.1186/s12889-018-5290-3 (PMC5861615; doi:10.1186/s12889-018-5290-3)
Supplement: Supplementary file 1 — Descriptive statistics of sample. Sample characteristics including gender, age, educational level, Index of Multiple Deprivation scores, ethnicity and employment status for all participants. (DOCX 20 kb) [file 12889_2018_5290_MOESM1_ESM.docx]

**Additional file 1**

**Descriptive statistics of sample**

| ***Table A.1. Children gender*** | | | | | | | |
| --- | --- | --- | --- | --- | --- | --- | --- |
|  | | | Total (n) | | Percent | |  |
|  | Male | 24 | | 48.0 | |  |  |
|  | Female | 26 | | 52.0 | |  |  |
|  | Total | 50 | | 100.0 | |  |  |

| ***Table A.2. Adults gender*** | | | | |
| --- | --- | --- | --- | --- |
|  | | Total (n) | Percent |  |
|  | Male | 11 | 22.0 |  |
|  | Female | 39 | 78.0 |  |
|  | Total | 50 | 100.0 |  |

| ***Table A.3. Age*** | | | | | |
| --- | --- | --- | --- | --- | --- |
|  | Total (n) | Minimum age | Maximum age | Mean  age | Std.  Deviation |
| Adults | 50 | 26 | 54 | 34.62 | 5.030 |
| Children | 50 | 3.00 | 5.70 | 4.2882 | .88074 |

| ***Table A.4. Index of Multiple Deprivation (IMD) scores (refers to family in general including adults and children)*** | | | | | | | | | | | |
| --- | --- | --- | --- | --- | --- | --- | --- | --- | --- | --- | --- |
| IMD | | | Total (n) | | | | Percent | | |  |  |
|  | 2^nd^ quintile | 5 | | | | 10.0 | | | |  |  |
|  | 3^rd^ quintile | 11 | | | | 22.0 | | | |  |  |
|  | 4^th^ quintile | 13 | | | | 26.0 | | | |  |  |
|  | 5^th^ quintile | 21 | | | | 42.0 | | | |  |  |
|  | Total | 50 | | | | 100.0 | | | |  |  |
| ***Table A.5. Adults education level*** | | | | | | | | | | | |
| Education level | | | | | Total (n) | | | | Percent | |  |
|  | Postgraduate | | | 2 | | | | 4.0 | | |  |
|  | University | | | 13 | | | | 26.0 | | |  |
|  | Post-High School (no University) | | | 21 | | | | 42.0 | | |  |
|  | Up to High School | | | 14 | | | | 28.0 | | |  |
|  | Total | | | 50 | | | | 100.0 | | |  |

| ***Table A.6. Ethnicity (refers to family in general including adults and children)*** | | | | | | | | | | | |
| --- | --- | --- | --- | --- | --- | --- | --- | --- | --- | --- | --- |
| Ethnicity | | | | Total (n) | | | Percent | | |  |  |
|  | | White | 33 | | | 66.0 | | |  |  |  |
|  |  | Asian | 9 | | | 18.0 | | |  |  |  |
|  |  | Black | 8 | | | 16.0 | | |  |  |  |
|  |  | Total | 50 | | | 100.0 | | |  |  |  |
| ***Table A.7. Adults Employment status*** | | | | | | | | | | | |
|  | | | | | Total (n) | | | Percent | | |  |
|  | Full-Time | | | | 5 | | | 10.0 | | |  |
|  | Part-Time | | | | 19 | | | 38.0 | | |  |
|  | Self-Employed | | | | 5 | | | 10.0 | | |  |
|  | Stay at home parent | | | | 21 | | | 42.0 | | |  |
|  | Total | | | | 50 | | | 100.0 | | |  |
